# Mortality from diabetes mellitus and its impact on life expectancy at 60 years of age in Mexico

**DOI:** 10.11606/s1518-8787.20210550032929

**Published:** 2021-10-18

**Authors:** María Guadalupe Vega-López, Guillermo Julián González-Pérez

**Affiliations:** I Universidad de Guadalajara Centro Universitario de Ciencias de la Salud Centro de Estudios en Salud, Población y Desarrollo Humano GuadalajaraJAL México Universidad de Guadalajara. Centro Universitario de Ciencias de la Salud. Centro de Estudios en Salud, Población y Desarrollo Humano. Guadalajara, JAL, México

**Keywords:** Elderly, Diabetes Mellitus, mortality, Mortality, trends, Life Expectancy

## Abstract

**OBJECTIVES:**

To analyze the behavior of mortality from diabetes mellitus (DM) for both sexes in Mexico from 1998 to 2018, and its impact on life expectancy (LE) from 60 to 85 years of age in the three-year periods 1998–2000 and 2016–2018, compared with other causes of death, as well as to determine the loss of years of life expectancy associated with DM in each three-year period.

**METHODS:**

The current study is observational and descriptive. Age-adjusted rates of mortality from DM were calculated for each sex from 1998 to 2018. Sex-specific life tables were constructed for 1998–2000 and 2016–2018, and both LE between 60 and 85 years, and years of life expectancy lost (YLELL) due to DM and selected causes between both ages were calculated.

**RESULTS:**

Between 1998 and 2018, the adjusted DM-resulting male mortality rate grew 55% in the population aged 60 and over, while the female mortality rate grew 20%. Between 1998–2000 and 2016–2018, male LE for 60–85 age group decreased 0.22 years, while female LE increased 0.24. In 2016-2018, DM was responsible for 1.30 YLEL among men of 60 to 85 years (19% of the total YLEL), and 1.24 YLEL for women (24% of the total), more than the other causes analyzed.

**CONCLUSIONS:**

The increase in mortality from DM has substantially contributed both to reduce LE of older adult men, and to slow the increase of LE among women aged 60 years and older so far this century. Thus, preventive policies should be implemented since early ages to reduce the high levels of overweight and obesity in the country and, therefore, the significant population ratio suffering from DM.

## INTRODUCTION

According to the World Health Organization (WHO), diabetes mellitus (DM) is among the leading causes of death on a global scale^1^. In North America and the Caribbean, in 2019 it accounted for 13.8% of deaths from all causes in the age group of 20 to 79 years. In that same year, Mexico had estimated 12.8 million people suffering from DM. This figure placed it in sixth place worldwide among the countries with the highest number of adults with the disease^2^. In 20 years, the mortality rate from DM in Mexico rose from 43.4 to 80.1 per 100,000 inhabitants (1,563,896 deaths between 1998 and 2018), i.e., it almost doubled^3^. DM has contributed as the first and second causes of death in the 45 to 64 and 65 and older age groups, respectively^4^.

In fact, the growth in deaths due to non-communicable diseases (NCDs), including DM, coupled with the high rates of violent deaths, have slowed the increase of life expectancy (LE) of the Mexican population. While for the Region of the Americas, LE at birth increased from 73.6 to 76.8 between 2000 and 2016^1^ (an increase of 3.2 years), in Mexico the increase was only 2 years^5^.

In accordance with the objective known as “25 X 25” of the WHO Global Action Plan 2013–2020 for the reduction of mortality between 30 and 70 years of age^6^, WHO estimated that if the risk factors that most affect DM and the other three NCDs (cardiovascular diseases, cancer, and chronic respiratory diseases) were controlled by 2025, premature mortality would decrease by 25%. However, nations such as Mexico would have serious problems in intervening on risk factors^7^ and, therefore, the impact of DM on the evolution of LE would continue to be notable.

Several authors have studied the effect of mortality from DM on life expectancy, either at birth or starting in a specific age, in different countries^8^. However, there are few studies in Mexico that analyze the impact of DM on the LE of older adults in the last 20 years^11^.

Thus, in view of the relevance attained by DM, especially at advanced ages, the current study aims to analyze the behavior of mortality from this cause for both sexes at national level between 1998 and 2018, and the impact of DM mortality on life expectancy in the age group of 60 to 85 years in the three-year periods 1998–2000 and 2016–2018 in comparison with other causes of death. Moreover, it intends to determine the DM-associated loss of years of life expectancy in each three-year period.

## METHODS

This is an observational and descriptive study, based on secondary sources of information and focused on the older adult population (persons of 60+ years). Mortality data were extracted from the official databases of deaths (*Cubos Dinámicos*) of the General Health Information Board of the Health Secretariat^12^. Information on population for the years reviewed was obtained from the estimates and forecasts of the National Population Council (CONAPO)^13^.

In particular, DM and the other nine causes of death studied, considered to be among the leading causes of death for the population aged 60 years and older, were classified according to the International Classification of Diseases (ICD-10): DM; homicide; suicide; motor vehicle traffic accidents (MVA); ischemic heart disease; malignant tumors; cerebrovascular diseases; hepatic cirrhosis and other liver diseases; chronic obstructive pulmonary disease (COPD); and calorie-protein malnutrition.

Using existing data on death and population, age-adjusted DM mortality rates were calculated using the direct method for both the general population and those aged 60 years and older, by sex, between 1998 and 2018. For that, DM mortality rates were initially calculated by five-year age and sex groups for each year of the period under analysis. The standard population was the total population of Mexico in 2018 by five-year age groups, to calculate the adjusted rates in the general population. The total population aged 60 years and over in Mexico in 2018 by five-year age groups was used to calculate the adjusted rates in older adult population.

In turn, by means of the joinpoint regression analysis, using the Joinpoint software, version 4.8.0.1^14^, we estimated the average annual percentage change in adjusted rates and their statistical significance (p-value < 0.05) by sex, for both the general population and the population aged 60 years and over, between 1998 and 2018. This indicator is a summary measure of the trend during a prespecified time interval, such as the one analyzed herein. It should be noted that joinpoint regression models, described in detail in the literature^15^ identify the points that disclose significant changes in the trend and, in addition, estimate the trend observed in the period of review. Therefore, they provide better fit when compared to linear regression models, which reduce the trend to a single equation.

Abridged life tables were also built at national level for both sexes for each of the three-year periods studied, using the EPIDAT v4.2^18^ software. The three-year periods of 1998–2000 and 2016–2018 were elected as these allow comparing the most recent situation in the country for which mortality information is available with that existing almost 20 years earlier, in addition to enable working with data referring exclusively to ICD-10. Likewise, the construction of triennial mortality tables intended to reduce possible random variations in mortality that could bias the trend of its behavior.

Based on the life tables, and in accordance with Arriaga’s method^19^ (described in detail in the literature^20,21^), we calculated both the temporary life expectancy (TLE) between 60 and 85 years, and the years of life expectancy lost (YLEL) between both ages (in general, for DM and the other causes analyzed, and only for DM, by age group in each three-year period). The YLEL were calculated by multiplying the ratio of persons who die between ages *x* y *x* + *n* in the stationary population of the life table, by means of the difference between the average number of years they could have lived from *x* if they had not died, and the average number of years actually lived between *x* y *x* + *n* by the population that dies in that age group^19,20^. Both indicators were calculated using the EPIDAT v4.2^18^ software, assuming zero mortality between 60 and 85 years.

The research protocol “Mortality by causes in Jalisco and Mexico”, source of this article, was approved by the Center for Health, Population and Human Development Studies of the University of Guadalajara and registered under the code SyP- 2015-002. The project is developed in accordance with the ethical guidelines set out in the Regulations of the General Health Law on health research in Mexico, since it was considered as “risk-free research”, in that it only works with secondary data, using documentary research techniques and methods with protected data that do not affect any individual.

## RESULTS

Deaths from DM have increased in each sex between both three-year periods studied (Table 1), in a higher proportion than total deaths (157% vs. 60% among men, 115% vs. 58% among women). In particular, the proportional increase in the number of deaths due to DM has been greater in the population aged 60+ years (163% among men and 120% among women).

**Table 1:** Deaths, total and from diabetes mellitus (DM), overall and 60 years and older; General population and 60 years and older; mortality rates, general and from diabetes mellitus (per 100,000 population), by sex. Mexico, 1998–2000 and 2016–2018.

	1998–2000	2016–2018	Increase between both three-year periods (in %)
Men			
Total deaths	741,165	1,184,803	59.86
Deaths 60+	371,817	688,518	85.18
Deaths from DM	59,742	153,712	157.29
Deaths from DM 60+	41,042	107,901	162.90
Total population	147,442,948	180,676,918	22.54
Population 60+	10,425,356	17,937,931	72.06
Rate of death from DM	40.52	85.08	109.97
Rate of death from DM 60+	393.67	601.52	52.80
Women			
Total deaths	584,692	925,379	58.27
Deaths 60+	370,722	668,463	80.31
Deaths from DM	74,335	159,639	114.76
Deaths from DM 60+	55,987	123,249	120.14
Total population	151,644,354	189,852,618	25.20
Population 60+	11,534,759	20,998,824	82.05
Rate of death from DM	49.02	84.09	
Rate of death from DM 60+			

This increase in deaths from DM is reflected in mortality rates: the rate more than doubles among men in general and increases by 53% in older men; in women, increases are 72% and 21%, respectively (Table 1). The fact that mortality rates from DM in older adults increase less than those of the general population is related to the growth rate of the population aged 60 years and older, which in both sexes more than triples that of the general population (72% vs. 23% among men, 82% vs. 25% for women).

**Figure 1:**
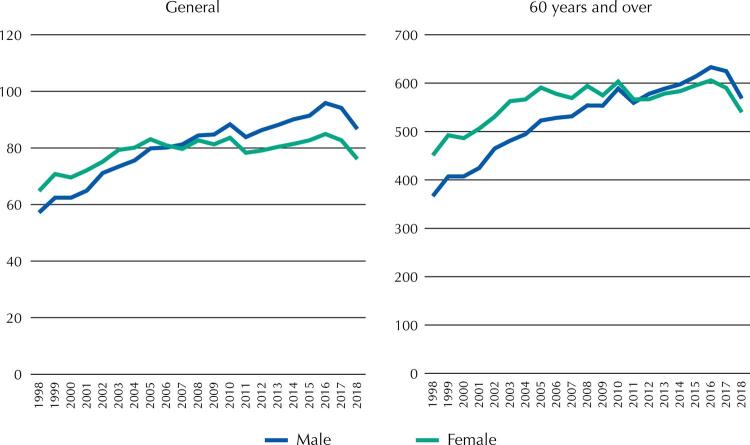
Adjusted rates of mortality from diabetes mellitus (per 100,000 inhabitants) to the general population and population of 60 years or over, by sex. Mexico, 1998–2018.

Analysis of the trend in the adjusted rates of mortality due to DM (Figure 1) reveals in both sexes, and for both the general population and the elderly, a notable increase in the rates during the period studied (except for the last year, when the rates fell slightly). However, the average annual percentage change in the adjusted rates was much greater for men than for women: in each population (general and 60 years and over), the estimated value of the average annual percentage change in male rates (2.1% and 2.2% for each case, both significantly different from 0) doubled that of female rates (1.1% and 1.1%, also significantly different from 0).

While the female rate at the beginning of the period was clearly higher than the male rate, with sharper difference in older adults, still in 2007 in the general population, and in 2012 in the older adult population, it was observed that the male rate exceeded the female rate, a trend that continued until the end of the period. In the last years of the period studied, male excess mortality from DM was higher in the general population than in the population aged 60 years and older.

Table 2 shows that the male TLE between 60 and 85 years decreased by 0.22 years between 1998–2000 and 2016–2018. A look at the YLEL based on causes of death shows that DM was the cause reporting the greatest increase in YLEL (0.399), and also the one that maintained higher annual growth rate between both dates (2.01%). From the third leading cause of death according to YLEL in 1998–2000, DM became the first in 2016–2018, overtaking malignant tumors and ischemic heart disease (although the YLEL for the latter also increased). In practice, it was responsible for almost one-fifth of YLEL among men in the last three-year period reviewed.

**Table 2:** Years of life expectancy lost (YLEL) by selected causes, and temporary life expectancy (TLE) from 60 to 85 years old. Male sex; Mexico, 1998–2000 and 2016–2018.

Cause			Absolute change	Relative annual change (%)	Percentage of the total YLEL 1998–2000	Percentage of the total YLEL 2016–2018
Homicide	0.0671	0.0684	0.0013	0.11	1.02	1.01
Suicide	0.0219	0.0229	0.0010	0.25	0.33	0.34
MVA	0.0434	0.0569	0.0135	1.50	0.66	0.84
Diabetes Mellitus	0.9054	1.3044	0.3990	2.01	13.82	19.28
Ischemic heart diseases	0.9476	1.1624	0.2148	1.13	14.46	17.18
Malignant tumors	0.9936	0.9990	0.0054	0.03	15.17	14.77
Cerebrovascular diseases	0.4539	0.3571	-0.0968	-1.33	6.93	5.28
Alzheimer	0.0088	0.0130	0.0042	2.14	0.13	0.19
Hepatic cirrhosis	0.5318	0.3059	-0.2259	-3.00	8.12	4.52
COPD	0.3398	0.2607	-0.0791	-1.46	5.19	3.85
Calorie-protein malnutrition	0.0967	0.0419	-0.0548	-4.39	1.48	0.62
Remaining causes	2.1410	2.1728	0.0318	0.08	32.68	32.12
Total YLEL	6.55	6.77	0.22	0.18		
Temporary life expectancy	18.45	18.23	-0.22			

MVA: motor vehicle traffic accidents; Dis.: diseases; COPD: chronic obstructive pulmonary disease.

Other relevant causes of death in the male older adult population, such as hepatic cirrhosis, COPD and cerebrovascular diseases, experienced a significant decrease in the number of YLEL, while malignant tumors remained practically at the same level in both three-year periods. The YLEL due to caloric-protein malnutrition had the largest decrease observed in the period (with numbers in 2016–2018 less than half of those calculated for 1998–2000), while the causes of violent death studied presented an almost marginal increase in YLEL and accounted for little more than 2% of the total number of YLEL from 60 to 85 years in each three-year period.

For women, YLEL between 60 and 85 years old presented an increase of just 0.24 years between 1998–2000 and 2016–2018 (Table 3). In each three-year period, DM led the causes studied according to the number of YLEL, in both cases exceeding one YLEL. In 2016-2018, it was responsible for almost a quarter of YLEL among women of 60 to 85 years. Likewise, it was the cause that increased the most in absolute numbers (0.1153). Only suicides reported growth rate in the number of YLEL higher than DM in the period analyzed, although the number of YLEL due to this cause was small.

**Table 3:** Years of life expectancy lost (YLEL) by selected causes, and temporary life expectancy (TLE) from 60 to 85 years old. Female sex; Mexico, 1998–2000 and 2016–2018.

Cause			Absolute change	Relative annual change (%)	Percentage of the total YLEL 1998–2000	Percentage of the total YLEL 2016–2018
Homicide	0.0121	0.0102	-0.0019	-0.95	0.22	0.20
Suicide	0.0023	0.0031	0.0008	1.65	0.04	0.06
MVA	0.0172	0.0181	0.0009	0.28	0.32	0.35
Diabetes Mellitus	1.1255	1.2408	0.1153	0.54	20.66	23.85
Ischemic heart diseases	0.6630	0.6964	0.0334	0.27	12.17	13.39
Malignant tumors	1.0152	0.9087	-0.1065	-0.62	18.64	17.47
Cerebrovascular diseases	0.4410	0.3032	-0.1378	-2.06	8.10	5.83
Alzheimer	0.0112	0.0167	0.0055	2.19	0.21	0.32
Hepatic cirrhosis	0.2243	0.1373	-0.0870	-2.67	4.12	2.64
COPD	0.2191	0.1896	-0.0295	-0.80	4.02	3.64
Calorie-protein malnutrition	0.0812	0.0321	-0.0491	-4.82	1.49	0.62
Remaining causes	1.6347	1.6454	0.0107	0.04	30.01	31.63
Total YLEL	5.45	5.20	-0.24	-0.26		
Temporary life expectancy	19.56	19.80	0.24			

MVA: motor vehicle traffic accidents; Dis.: diseases; COPD: chronic obstructive pulmonary disease.

Except for ischemic heart disease, where there was a slight increase in the number of YLEL between the two three-year periods, there was a significant reduction in YLEL for other major causes, such as cerebrovascular disease, hepatic cirrhosis, and COPD. This was even sharper in the case of calorie-protein malnutrition. Similarly, there was a reduction in the number of deaths due to homicide. In general, the violent deaths analyzed did not account for even 1% of the total number of YLEL in either of the two three-year periods.

**Figure 2:**
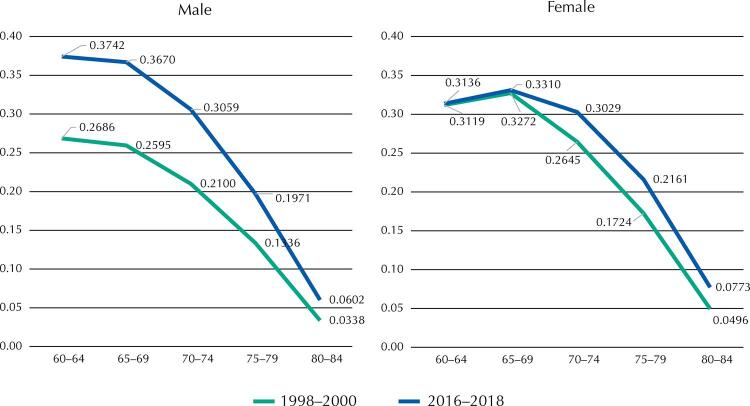
Years of life expectancy lost (YLEL) between 60 and 85 years of age from diabetes mellitus, by groups of age and sex. Mexico, 1998–2000 and 2016–2018.

Finally, the analysis of YLEL due to DM by age group in the older adult population (Figure 2) allowed us to identify that, in the case of men, YLEL increased in all ages between 1998–2000 and 2016–2018, with the highest values found in the 60-64 years age group. In both three-year periods, YLEL among men of 60 to 69 years accounted for a similar proportion of the total YLEL: 58% in 1998–2000 and 57% in 2016–2018.

In contrast to men, the number of DM-related YLEL among women of 60 to 69 years of age reported virtually no increase in the period studied, with a moderate increase observed from the age of 70 years onward. The highest number of YLEL for each three-year period was in the 65–69 years age group. While in 1998–2000 the YLEL between 60 and 69 years accounted for about 57% of the total number of YLEL from 60 to 85 years; in 2016–2018 that ratio decreased to 52%.

## DISCUSSION

According to WHO^5^, LE at age 60 has not increased in the same way in Latin America. Countries with structural base and socio-political relations similar to that of Mexico, which in 2000 reported a LE at age 60 similar to or even slightly lower than Mexico (20.1 years for men and 22.9 for women), have made greater progress in LE at that age: while in 2016 the figures showed an increase in LE at age 60 in Mexico of 0.9 years for men and 0.6 for women, in Chile the increase was 2.0 and 1.6 respectively; in Costa Rica, 2.0 and 1.7; in Colombia, 2.0 and 2.3; and in Brazil, 3.0 and 2.9.

In accordance with the foregoing, the results found in this study reveal the stagnation of the increase in LE among Mexican older adults: the TLE between 60 and 85 years of age barely grew in the case of women and even slightly decreased for men between the three-year periods of 1998–2000 and 2016–2018.

In this regard, the analysis of the YLEL by cause highlights that DM was the cause not only of the highest number of YLEL between 60 and 85 years in both sexes in each three-year period (accounting for one-fifth of all YLEL among men, and almost one-quarter among women in 2016–2018), but also the one in which YLEL had the highest rate of growth among the causes of highest mortality in older adults. This result is more transcendent in view of the above-mentioned stagnation of LE at age 60.

These findings are undoubtedly related to the sharp increase in the adjusted mortality rate due to DM for both sexes, even sharper among men than among women, during the period studied both in the general population and among older adults. The increase in rates in Mexico for the 60+ group (55% for men, 20% for women between 1998 and 2018) was higher than that observed in countries of the continent such as Brazil and Chile between 1999 and 2015, while in others (Colombia, Costa Rica) a reduction in the rate was even observed in the same period^22^.

In turn, the notable increase in male mortality rate due to DM is reflected in the fact that, in opposition to 1998–2000, in 2016–2018 the YLEL for men between 60 and 85 years of age due to this cause (1.30) exceeded those of women (1.24). In this sense, it is notorious that as DM-related deaths of men increase at relatively younger ages than women (between 60 and 69 years), more years of life are lost.

Using different methodologies, several studies have analyzed the effect of DM on LE. A recent study in the United States estimated the YLEL for men pf 30 years of age at 0.83, and 0.89 for women, rising to 1.05 years for black women^9^. In Scotland, a study carried out in a population with and without type II DM from different socioeconomic strata showed a significant reduction in life expectancy in both sexes (greater among women) and in almost all socioeconomic strata in any age segment analyzed^10^. In Mexico, Davila and Pardo examined the effect of DM on the change in YLEL between 1990–2000 and 2000–2010, observing 0.32 and 0.12 YLEL among women for each period, and 0.31 and 0.34 among men^11^. In another study comparing YLEL between 20 and 79 years in Colombia and Mexico, they found that for Colombia YLEL went from 0.35 in 1998 to 0.33 in 2007 and in Mexico, on the contrary, it increased from 0.85 to 1.1^23^.

Both the demographic and epidemiological transitions have a clear influence on the behavior of DM reviewed in this study. Not only has the population aged 60 years and older, more prone to DM and other NCDs, notably increased, but mortality from this cause has also increased substantially. Comorbidity is not exclusive to the elderly, although in this group it increases the probability of death. The current study shows that two causes commonly associated, namely DM and ischemic heart disease, had the greatest impact on the loss of years of life in both sexes.

The risk of developing DM (frequently coupled with other NCDs) exposes the person to a series of organic complications^24^ as well as family and economic difficulties^27,28^, and is more acute in older adults. For the family, it means concentrating material and psychological support on the patient, and possibly direct expenses related to treatment and hospitalization costs. For countries with low to medium economic level, the burden of NCDs implies an expense that may exceed their capacity to cope with. DM-related expenses in Mexico reached 17.0 billion dollars in 2019^2^, posing an economic and a health challenge.

Two of the main risk factors for developing DM have had a particularly high impact in Mexico: obesity and physical inactivity. Excess weight is observed in the population over 60 years of age, especially in the 60–69 years age group, where 54% of the population is overweight and 14.3% is obese^29^. At the international level, it has been found that obesity in the population aged 55 years and over is associated with a reduction in the number of years lived free of diabetes^8^. Regarding vigorous physical activity, the age group of 20 to 60 years engages less than 150 minutes per week is exercises^29^. Particularly in the 55+ group, 60.1% of men and 68.7% of women have declared to be inactive^30^.

The importance of lifestyle change in addressing the DM epidemic has been considered. Although educational interventions may succeed in the medium term^31^, local studies in Mexico have shown that changes are observed after an intervention, but the optimal reduction in clinical and biochemical parameters in diabetic patients is not achieved^32,33^. In addition to the so-called “Hawthorne effect”, it should be considered that changes in behavior are not linear, comprising at least two components: life choices and life opportunities. The individual’s range of freedom to choose is confronted with his or her life situation. Structural constraints may be dominant. Attach all responsibility to the individual may be inappropriate and unfair.

The current study may have some limitations, mostly related to the collection of mortality data. The coverage and quality of mortality registries in Mexico has been considered satisfactory by the Pan American Health Organization^34^ and has improved in recent years^35^, clearly supporting the consistency of the data used. Even so, it is worth noting that there may be difficulties in the coding of DM as a basic cause of death, mainly because individuals with the disease die from complications associated with cardiovascular or renal diseases, for example, which could to some extent distort the true dimension of the problem^23,36^. This situation would deserve, per se, more detailed investigation. This, however, goes beyond the objectives of the present study.

Regarding the method used to calculate the YLEL, the use of the assumption of zero mortality among three possible options does not alter the meaning of the results and is recommended by literature^21^ as it facilitates the interpretation of the results, and fully explains the changes in the TLE.

Notwithstanding the aforementioned limitations, the results found allow a reasonable approximation of the recent behavior of mortality from DM in older adults in Mexico. Considering the implications of this research’s findings that DM is substantially reducing the LE of the Mexican population, especially in older adults and more sharply among men, it would be advisable to implement (or intensify) preventive measures against DM from an early age, at the mere suspicion of its potential onset, and not only focusing on those who have been diagnosed. In this regard, effective public policies should be generated to promote healthy lifestyles, such as those aimed at reducing the consumption of sugar-sweetened beverages, and those that favor an adequate diet in schools.

Likewise, it is essential to allow patients to express their points of view, from a proactive and co-responsible standing, giving the opportunity to identify the social determinants that restrict the possibility of permanent changes in their lifestyles, to seek possible solutions and thus timely avoid deterioration of health, onset of comorbidities and increased costs of care, both for the population and for the health system.
