# Supplementary material for: Mortality from diabetes mellitus and its impact on life expectancy at 60 years of age in Mexico
Source: Rev Saude Publica. 2021 Oct 18;55:61. doi: 10.11606/s1518-8787.20210550032929 (PMC8522739; doi:10.11606/s1518-8787.20210550032929)
Supplement: Translation

# Translation: Mortalidad por diabetes mellitus y su impacto en la esperanza de vida a los 60 años en México

**Authors:** María Guadalupe Vega-López, Guillermo Julián González-Pérez

**Affiliations:** I México Universidad de Guadalajara. Centro Universitario de Ciencias de la Salud. Centro de Estudios en Salud, Población y Desarrollo Humano. Guadalajara, JAL, México

**Keywords:** Anciano, Diabetes Mellitus, mortalidad, Mortalidad, tendencias, Esperanza de Vida

## Abstract

**OBJETIVOS:**

Analizar el comportamiento de la mortalidad por diabetes mellitus (DM) para ambos sexos en México entre 1998 y 2018 y su impacto sobre la esperanza de vida (EV) entre los 60 y 85 años de edad en los trienios 1998–2000 y 2016–2018 – en comparación con otras causas de muerte –, así como determinar la pérdida de años de esperanza de vida asociados a la DM en cada trienio.

**MÉTODOS:**

El presente estudio es observacional y descriptivo. Se calcularon tasas de mortalidad por DM ajustadas por edad para cada sexo entre 1998 y 2018. Se construyeron tablas de vida por sexo para 1998–2000 y 2016–2018 y se calculó tanto la EV entre 60 y 85 años, como los años de esperanza de vida perdidos (AEVP) por DM, y causas seleccionadas, entre ambas edades.

**RESULTADOS:**

Entre 1998 y 2018 la tasa ajustada de mortalidad masculina por DM creció 55% en la población de 60 y más, y la femenina 20%. Entre 1998–2000 y 2016–2018, la EV masculina entre 60 y 85 años se redujo 0,22 años, en tanto la femenina aumentó 0,24. En 2016–2018 la DM fue responsable, para los hombres, de 1,30 AEVP entre 60 y 85 años, (19% del total de AEVP) y para las mujeres 1,24 AEVP (24% del total), más que el resto de causas analizadas.

**CONCLUSIONES:**

El incremento de la mortalidad por DM ha contribuido de manera sustancial tanto a reducir la EV de los hombres adultos mayores, como a ralentizar el aumento de la EV de las mujeres de 60 años y más en lo que va de siglo. Así, resulta necesario implementar políticas preventivas desde edades tempranas que permitan reducir los altos niveles de sobrepeso y obesidad existentes en el país, y por ende, la notable proporción de población que padece DM.

## INTRODUCCIÓN

De acuerdo con la Organización Mundial de la Salud (OMS), entre las primeras causas de muerte en escala global figura la diabetes mellitus (DM)^1^. En América del Norte y el Caribe representó en 2019 el 13,8% de las muertes por todas las causas entre los 20 y 79 años. En ese mismo año, en México se estimó que 12.8 millones de personas padecían DM, cifra que lo colocó en el sexto lugar a nivel mundial entre los países con mayor cantidad de adultos con la enfermedad.^2^ En un lapso de 20 años, la tasa de mortalidad por DM pasó en México de 43,4 a 80,1 por 100 000 habitantes (1.563.896 decesos entre 1998 y 2018), esto es, casi se duplicó^3^. La DM ha contribuido como primera y segunda causa de muerte en los grupos de edad de 45 a 64 y de 65 y más años respectivamente^4^.

De hecho, el crecimiento de las defunciones por enfermedades no transmisibles (ENT), obviamente la DM entre ellas, sumado a las elevadas tasas de muertes violentas, han ralentizado el incremento en la esperanza de vida (EV) de la población mexicana. Mientras que para la Región de las Américas la EV al nacer pasó de 73,6 a 76,8 entre 2000 y 2016^1^ (un incremento de 3,2 años), en México el aumento fue solo de 2 años^5^.

De acuerdo con el objetivo conocido como “25 X 25” del Plan de Acción Global 2013–2020 de la OMS para la reducción de mortalidad entre los 30 y 70 años de edad^6^, la OMS estimó que, si para el año 2025 se controlaran los factores de riesgo que más afectan a la DM y otras tres ENT (enfermedades cardiovasculares, cáncer y enfermedades respiratorias crónicas), la mortalidad prematura disminuiría en un 25%. Sin embargo, naciones como México tendrían serios problemas para incidir sobre los factores de riesgo^7^ y, por tanto, el impacto de la DM en la evolución de la EV continuaría siendo notable.

Diversos autores han estudiado el efecto de la mortalidad por DM en la esperanza de vida, ya sea al nacer o a partir de una edad exacta determinada, en diferentes países^8^; no obstante, en México son escasos los estudios que analizan el impacto de la DM en la EV de los adultos mayores en los últimos 20 años^11^.

Así, en atención a la relevancia alcanzada por la DM, sobre todo en edades avanzadas, en el presente estudio se pretende analizar el comportamiento de la mortalidad por dicha causa para ambos sexos a nivel nacional entre 1998 y 2018 y el impacto de la mortalidad por DM sobre la esperanza de vida entre los 60 y 85 años de edad en los trienios 1998–2000 y 2016–2018, en comparación con otras causas de muerte, así como determinar la pérdida de años de esperanza de vida asociados a la DM en cada trienio.

## MÉTODOS

Este es un estudio observacional y descriptivo, basado en fuentes secundarias de información y centrado en la población adulta mayor (personas de 60 años y más); los datos sobre mortalidad se extrajeron de las bases de datos oficiales de defunciones (Cubos Dinámicos) de la Dirección General de Información en Salud de la Secretaría de Salud^12^. La información sobre población para los años estudiados fue obtenida de las estimaciones y proyecciones del Consejo Nacional de Población (CONAPO)^13^.

En particular, la DM y las otras nueve causas de muerte estudiadas, consideradas entre las principales causas de defunción para la población de 60 años y más, se clasificaron de acuerdo con la Clasificación Internacional de Enfermedades (CIE-10): DM; homicidio; suicidio; accidentes de tráfico de vehículo motor (ATVM); enfermedades isquémicas del corazón; tumores malignos; enfermedades cerebrovasculares; cirrosis hepática y otras enfermedades del hígado; enfermedad pulmonar obstructiva crónica (EPOC); y desnutrición calórico-proteica.

Con los datos existentes sobre defunciones y población se calcularon mediante el método directo las tasas de mortalidad por DM ajustadas por edad tanto para la población general como para aquella de 60 años y más, por sexo, entre 1998 y 2018. Para ello, se calcularon inicialmente tasas de mortalidad por DM por grupos quinquenales de edad y sexo para cada año del lapso estudiado. Se consideró como población estándar a la población total de México en 2018 por grupos quinquenales de edad, para el cálculo de las tasas ajustadas en la población en general, y a la población total de 60 años y más de México en 2018 por grupos quinquenales de edad, para el cálculo de las tasas ajustadas en la población adulta mayor.

A su vez, mediante el análisis de regresión “*joinpoint*”, utilizando el programa Joinpoint, versión 4.8.0.1^14^, se estimó el cambio porcentual anual promedio de las tasas ajustadas y su significación estadística (valor p < 0,05), por sexo, tanto para la población general como para la de 60 años y más, entre 1998 y 2018. Este indicador es una medida resumida de la tendencia durante un intervalo preestablecido, como el analizado en este estudio. Cabe señalar que los modelos de regresión “joinpoint” descritos en detalle en la literatura^15^ identifican los puntos que revelan cambios significativos en la tendencia y, además, estiman la tendencia observada en el período analizado, por lo que brindan un mejor ajuste si se comparan con los modelos de regresión lineal, que reducen la tendencia a una sola ecuación.

Asimismo, se construyeron para ambos sexos tablas de mortalidad abreviadas a nivel nacional para cada uno de los trienios estudiados, empleando para ello el programa EPIDAT v4.2^18^. La elección de los trienios 1998–2000 y 2016–2018 respondió a la posibilidad de comparar la situación más reciente del país para la que hay información disponible de mortalidad con la existente casi 20 años atrás, además de poder trabajar con datos referidos solo a la CIE-10. Asimismo, la construcción de tablas de mortalidad trienales tuvo la finalidad de reducir posibles variaciones aleatorias de la mortalidad que pudieran distorsionar la tendencia de su comportamiento.

A partir de las tablas de mortalidad, y de acuerdo con el método de Arriaga^19^ (descrito detalladamente en la literatura^20,21^), se calcularon tanto la esperanza de vida temporaria (EVT) entre 60 y 85 años como los años de esperanza de vida perdidos (AEVP) entre las dos edades (en general, para la DM y el resto de causas analizadas y, solo para la DM, por grupos de edad en cada trienio). Los AEVP se calcularon multiplicando la proporción de personas que fallecen entre las edades *x* y *x* + *n* en la población estacionaria de la tabla de mortalidad, por la diferencia entre el número medio de años que podrían haber vivido desde *x* en caso de no fallecer y el promedio de años realmente vividos entre *x* y *x* + *n* por la población que muere en ese grupo de edad^19,20^. Ambos indicadores fueron calculados mediante el programa EPIDAT v4.2^18^, asumiendo una mortalidad nula entre 60 y 85 años.

El protocolo de investigación “Mortalidad por causas en Jalisco y México”, de donde emana este artículo, fue aprobado por el Centro de Estudios en Salud, Población y Desarrollo Humano de la Universidad de Guadalajara y registrado con la clave SyP- 2015–002. El proyecto se desarrolla en concordancia con los lineamientos éticos expuestos en el Reglamento de la Ley General de Salud en materia de investigación para la salud de México, pues se consideró como “investigación sin riesgo”, en tanto solo se trabaja con datos secundarios, utilizándose técnicas y métodos de investigación documental con datos protegidos que no afectan a ningún individuo.

## RESULTADOS

Las defunciones debidas a DM se han incrementado en cada sexo entre ambos trienios estudiados (Tabla 1), en una proporción mayor que las defunciones totales (157% vs. 60% en los hombres, 115% vs. 58% en las mujeres); en particular, el incremento proporcional en el número de defunciones por DM ha sido mayor en la población de 60 años y más (163% en el sexo masculino y 120% en el femenino).

**Tabla 1:** Defunciones, total y por diabetes mellitus (DM), en general y de 60 años y más; Población general y de 60 y más; tasas de mortalidad, general y por diabetes mellitus (por 100.000 habitantes), según sexo. México, 1998–2000 y 2016–2018.

	1998–2000	2016–2018	Incremento entre ambos trienios (en %)
Hombres			
Defunciones totales	741.165	1.184.803	59,86
Defunciones 60+	371.817	688.518	85,18
Defunciones por DM	59.742	153.712	157,29
Defunciones por DM 60+	41.042	107.901	162,90
Población total	147.442.948	180.676.918	22,54
Población 60+	10.425.356	17.937.931	72,06
Tasa de mortalidad por DM	40,52	85,08	109,97
Tasa de mortalidad por DM 60+	393,67	601,52	52,80
Mujeres			
Defunciones totales	584.692	925.379	58,27
Defunciones 60+	370.722	668.463	80,31
Defunciones por DM	74.335	159.639	114,76
Defunciones por DM 60+	55.987	123.249	120,14
Población total	151.644.354	189.852.618	25,20
Población 60+	11.534.759	20.998.824	82,05
Tasa de mortalidad por DM	49,02	84,09	71,54
Tasa de mortalidad por DM 60+	485,38	586,93	20,92

Este aumento de las muertes a causa de la DM se refleja en las tasas de mortalidad: la tasa más que se duplica entre los hombres en general y se incrementa en 53% en los hombres adultos mayores; en las mujeres, los incrementos son de 72% y 21% respectivamente (Tabla 1). Que las tasas de mortalidad por DM en los adultos mayores aumenten en menor medida que las de la población en general está relacionado con el ritmo de crecimiento de la población de 60 años y más, que en ambos sexos más que triplica al de la población en general (72% vs. 23% en el caso de los hombres, 82% vs. 25% en el de las mujeres).

El análisis de la tendencia de las tasas ajustadas de mortalidad por DM (Figura 1) revela en ambos sexos, y tanto para la población en general como para los adultos mayores, un notable incremento de las tasas en el lapso estudiado (salvo el último año, cuando las tasas cayeron ligeramente); ahora bien, el cambio porcentual anual promedio de las tasas ajustadas fue mucho mayor en el caso de los hombres que de las mujeres: en cada población (general y 60 años y más), el valor estimado del cambio porcentual anual promedio de las tasas masculinas (2,1% y 2,2% para cada caso, ambos significativamente diferentes de 0) duplicó el de las tasas femeninas (1,1% y 1,1%, también significativamente diferentes de 0).

**Figura 1:** Tasas ajustadas de mortalidad por diabetes mellitus (por 100.000 habitantes), para población general y de 60 años y más, según sexo. México, 1998–2018.

Mientras que la tasa femenina al inicio del período fue claramente mayor a la masculina, con una diferencia más marcada en los adultos mayores, ya en 2007 en la población general y en 2012 en la población adulta mayor se observó que la tasa masculina rebasó la femenina, tendencia que se mantuvo hasta el final del período. En los últimos años del lapso estudiado, la sobremortalidad masculina por DM fue mayor en la población general que en la población de 60 años y más.

En la Tabla 2, se puede apreciar que la EVT masculina entre 60 y 85 años se redujo en 0,22 años entre 1998–2000 y 2016–2018. Una mirada a los AEVP según causas de muerte permite constatar que la DM fue la causa en la cual hubo un mayor aumento en los AEVP (0,399) y también la que mantuvo un ritmo de crecimiento anual mayor entre ambas fechas (2,01%). De ser la tercera causa de defunción según los AEVP provocados en 1998–2000, la DM se convirtió en 2016–2018 en la primera, desplazando a los tumores malignos y las enfermedades isquémicas del corazón (aunque esta última también incrementó el número de AEVP), y en la práctica, fue responsable de casi la quinta parte de los AEVP por los hombres en el último trienio analizado.

**Tabla 2:** Años de esperanza de vida perdidos (AEVP) por causas seleccionadas y esperanza de vida temporaria (EVT), entre 60 y 85 años. Sexo masculino; México, 1998–2000 y 2016–2018.

Causa	1998–2000	2016–2018	Cambio absoluto	Cambio relativo anual (%)	Porcentaje del total de AEVP 1998–2000	Porcentaje del total de AEVP 2016–2018
						
Homicidio	0,0671	0,0684	0,0013	0,11	1,02	1,01
Suicidio	0,0219	0,0229	0,0010	0,25	0,33	0,34
ATVM	0,0434	0,0569	0,0135	1,50	0,66	0,84
Diabetes mellitus	0,9054	1,3044	0,3990	2,01	13,82	19,28
Enf. isquémicas del corazón	0,9476	1,1624	0,2148	1,13	14,46	17,18
Tumores malignos	0,9936	0,9990	0,0054	0,03	15,17	14,77
Enf. cerebrovasculares	0,4539	0,3571	-0,0968	-1,33	6,93	5,28
Alzheimer	0,0088	0,0130	0,0042	2,14	0,13	0,19
Cirrosis hepática	0,5318	0,3059	-0,2259	-3,00	8,12	4,52
EPOC	0,3398	0,2607	-0,0791	-1,46	5,19	3,85
Desnutrición calórico-proteica	0,0967	0,0419	-0,0548	-4,39	1,48	0,62
Resto de causas	2,1410	2,1728	0,0318	0,08	32,68	32,12
Total de AEVP	6,55	6,77	0,22	0,18		
Esperanza de vida temporaria	18,45	18,23	-0,22			

ATVM: accidentes de tráfico de vehículo motor; Enf.: enfermedades; EPOC: enfermedad pulmonar obstructiva crónica.

Otras causas de muerte relevantes en la población adulta mayor masculina, como la cirrosis hepática, las EPOC y las enfermedades cerebrovasculares, experimentaron un importante descenso en el número de AEVP, mientras que los tumores malignos se mantuvieron prácticamente al mismo nivel en ambos trienios; los AEVP por desnutrición calórico-proteica tuvieron la mayor disminución observada en el período (con una cifra en 2016–2018 menor a la mitad de lo calculado para 1998–2000), mientras que las causas de muerte violenta estudiadas presentaron un aumento casi marginal en los AEVP y apenas representaron algo más del 2% del total de AEVP entre 60 y 85 años en cada trienio.

En el caso de las mujeres, la EVT entre 60 y 85 años mostró un aumento de apenas 0,24 años entre 1998–2000 y 2016–2018 (Tabla 3). La DM encabezó en cada trienio a las causas estudiadas según el número de AEVP, rebasando en ambos la cifra de un AEVP y siendo en 2016–2018 responsable de casi la cuarta parte de los AEVP femeninos entre 60 y 85 años; igualmente, fue la causa que más aumentó en números absolutos (0,1153). Solo los suicidios presentaron un ritmo de crecimiento mayor en el número de AEVP que la DM en el lapso analizado, aunque el número de AEVP por esta causa fue reducido.

**Tabla 3:** Años de esperanza de vida perdidos (AEVP) por causas seleccionadas y Esperanza de vida temporaria (EVT), entre 60 y 85 años. Sexo femenino; México, 1998–2000 y 2016–2018.

Causa	1998–2000	2016–2018	Cambio absoluto	Cambio relativo anual (%)	Porcentaje del total de AEVP 1998–2000	Porcentaje del total de AEVP 2016–2018
						
Homicidio	0,0121	0,0102	-0,0019	-0,95	0,22	0,20
Suicidio	0,0023	0,0031	0,0008	1,65	0,04	0,06
ATVM	0,0172	0,0181	0,0009	0,28	0,32	0,35
Diabetes mellitus	1,1255	1,2408	0,1153	0,54	20,66	23,85
Enf. isquémicas del corazón	0,6630	0,6964	0,0334	0,27	12,17	13,39
Tumores malignos	1,0152	0,9087	-0,1065	-0,62	18,64	17,47
Enf. cerebrovasculares	0,4410	0,3032	-0,1378	-2,06	8,10	5,83
Alzheimer	0,0112	0,0167	0,0055	2,19	0,21	0,32
Cirrosis hepática	0,2243	0,1373	-0,0870	-2,67	4,12	2,64
EPOC	0,2191	0,1896	-0,0295	-0,80	4,02	3,64
Desnutrición calórico-proteica	0,0812	0,0321	-0,0491	-4,82	1,49	0,62
Resto de causas	1,6347	1,6454	0,0107	0,04	30,01	31,63
Total de AEVP	5,45	5,20	-0,24	-0,26		
Esperanza de vida temporaria	19,56	19,80	0,24			

ATVM: accidentes de tráfico de vehículo motor; Enf.: enfermedades; EPOC: enfermedad pulmonar obstructiva crónica.

Salvo las enfermedades isquémicas del corazón, en las cuales se observó un ligero incremento en el número de AEVP entre ambos trienios, en el resto de las causas con un mayor peso, como las enfermedades cerebrovasculares, cirrosis hepática y las EPOC, se apreció una importante reducción en los AEVP; esta fue más marcada todavía en el caso de la desnutrición calórico-proteica. Asimismo, se redujeron los AEVP por homicidio. En general, las muertes violentas analizadas no representaron en ninguno de los dos trienios ni el 1% del total de AEVP.

Finalmente, el análisis de los AEVP a causa de la DM por grupos de edad en la población adulta mayor (Figura 2) permitió identificar que, en el caso de los hombres, los AEVP aumentaron en todas las edades entre 1998–2000 y 2016–2018, encontrándose los valores más altos en el grupo de 60–64 años. En ambos trienios, los AEVP masculinos entre 60 y 69 años representaron una proporción similar del total de AEVP: 58% en 1998-2000 y 57% en 2016–2018.

**Figura 2:** Años de esperanza de vida perdidos (AEVP) entre 60 y 85 años por diabetes mellitus, según grupos de edad y sexo. México, 1998–2000 y 2016–2018.

A diferencia de los hombres, en las mujeres la cifra de AEVP por DM entre 60 y 69 años prácticamente no aumentó en el período estudiado, observándose un incremento moderado a partir de los 70 años; el mayor número de AEVP se ubicó en ambos trienios en el grupo de 65 a 69 años. Mientras en 1998–2000 los AEVP entre 60 y 69 años representaron alrededor del 57% del total de AEVP entre 60 y 85 años, en 2016–2018 esa proporción se redujo a 52%.

## DISCUSIÓN

De acuerdo con la OMS^5^, la EV a los 60 años no ha aumentado de forma similar en Latinoamérica; países con una base estructural y de relaciones socio-políticas similares a México, que en el año 2000 reportaban una EV a los 60 años semejante o incluso ligeramente inferior a la de México (20,1 años para los hombres y 22,9 para las mujeres), han logrado mayores avances en la EV a esa edad: mientras que en 2016 las cifras reflejaban un incremento de la EV a los 60 años en México de 0,9 años para los hombres y 0,6 para las mujeres, en Chile el aumento fue de 2,0 y 1,6 respectivamente; en Costa Rica de 2,0 y 1,7; en Colombia, de 2,0 y 2,3; y en Brasil, de 3,0 y 2,9.

En concordancia con lo anterior, los resultados obtenidos en el presente estudio revelan el estancamiento del incremento de la EV entre los adultos mayores mexicanos: la EVT entre 60 y 85 años apenas creció en el caso de las mujeres e inclusive se redujo ligeramente para los hombres entre los trienios 1998–2000 y 2016–2018.

En tal sentido, el análisis de los AEVP por causas pone de relieve que la DM fue la causa que no solo provocó la cifra más elevada de AEVP entre 60 y 85 años en ambos sexos en cada trienio (representando la quinta parte de todos los AEVP por los hombres y casi la cuarta parte por las mujeres en 2016–2018), sino también aquella en la que los AEVP tuvieron un mayor ritmo de crecimiento entre las causas que ocasionan mayor mortalidad en los adultos mayores. Este resultado es más trascendente en vista del estancamiento previamente mencionado de la EV a los 60 años.

Estos hallazgos sin dudas están relacionados con el marcado crecimiento de la tasa ajustada de mortalidad por DM para ambos sexos –más acentuado en los hombres que en las mujeres- en el lapso estudiado, tanto en la población en general como entre los adultos mayores. El incremento de las tasas en México para el grupo de 60 y más (55% en el caso de los hombres, 20% en las mujeres entre 1998 y 2018) fue superior al observado en países del continente como Brasil y Chile entre 1999 y 2015, mientras que en otros (Colombia, Costa Rica) incluso se observó una reducción de la tasa en el mismo período^22^.

A su vez, el notable incremento de la tasa de mortalidad masculina por DM se ve reflejado en que a diferencia de 1998–2000, en 2016–2018 los AEVP por los hombres por esta causa entre 60 y 85 años (1,30) superaran a los de las mujeres (1,24); en tal sentido, es notorio el hecho de que al incrementarse las defunciones de hombres por DM en edades relativamente más tempranas que las mujeres (entre 60 y 69 años), se dejan de vivir más años.

Diversos estudios han analizado con diferentes metodologías el efecto de la DM en la EV; un estudio reciente en Estados Unidos estimaba en 0,83 los AEVP por los hombres a los 30 años, y 0,89 para las mujeres, cifra que se elevaba hasta 1,05 años para las mujeres negras^9^; en Escocia, una investigación realizada en población con y sin DM tipo II de diferentes estratos socioeconómicos evidenció una reducción importante en la esperanza de vida en ambos sexos (mayor entre las mujeres) y en casi todos los estratos socioeconómicos en cualquier segmento de edad analizado^10^; en México, Dávila y Pardo examinaron el efecto de la DM en el cambio de la EVT entre 1990–2000 y 2000–2010 observando 0,32 y 0,12 AEVP en mujeres en cada periodo y 0,31 y 0,34 en hombres^11^; en otro estudio comparando los AEVP entre 20 y 79 años en Colombia y México, encontraron que para Colombia los AEVP pasaron de 0,35 en 1998 a 0,33 en 2007 y en México, por el contrario, aumentaron de 0,85 a 1,1^23^.

Tanto la transición demográfica como la epidemiológica tienen franca influencia sobre el comportamiento de la DM analizado en esta investigación. No solo ha aumentado notablemente la población de 60 años y más, más propensa a padecer DM y otras ENT, sino que la mortalidad por dicha causa se ha incrementado de forma sustancial. La comorbilidad no es exclusiva de la gente mayor, aunque en ese grupo incrementa la probabilidad de muerte. El presente estudio evidencia que dos causas comúnmente asociadas, DM y enfermedades isquémicas del corazón, tuvieron la mayor repercusión en la pérdida de años de vida en ambos sexos.

El riesgo de desarrollar DM (frecuentemente junto a otras ENT a la vez), expone a la persona a enfrentar una serie de complicaciones orgánicas^24^, así como dificultades de índole familiar y económica^27,28^ y se agudiza en los adultos mayores. Para la familia significa concentrar ayuda material y psicológica en el enfermo y posiblemente un gasto directo invertidos en tratamiento y hospitalización. Para países con un nivel económico de bajo a medio la carga de ENT implica un gasto que puede rebasar su capacidad para afrontarlo. El gasto relacionado con la DM en México ascendió a 17.0 mil millones de dólares en 2019^2^, lo cual significa un reto económico y sanitario.

Dos de los principales factores de riesgo para desarrollar DM han tenido particularmente un alto impacto en México: obesidad e inactividad física. En el grupo de población mayor a 60 años hay exceso de peso, sobre todo en el grupo de 60 a 69 años, donde 54% de la población presenta sobrepeso y 14,3 % obesidad^29^. A nivel internacional, se ha encontrado que la obesidad en población de 55 años y más está asociada con una reducción en el número de años vividos libre de diabetes^8^. En cuanto a la actividad física vigorosa, entre los 20 y 60 años se practica menos de 150 minutos por semana^29^, y en particular en el grupo de 55 y más años, 60,1% de hombres y 68,7% de mujeres se han declarado inactivos^30^.

Se ha ponderado la importancia en el cambio de estilo de vida para enfrentar la epidemia de DM. Aunque intervenciones educativas pueden ser exitosas a mediano plazo^31^, estudios locales en México han evidenciado que tras una intervención se aprecian cambios pero no se llega a la disminución óptima en parámetros clínicos y bioquímicos en pacientes diabéticos^32,33^. Además del llamado “Efecto Hawthorne”, hay que tomar en cuenta que las modificaciones en el comportamiento no son lineales; este tiene al menos dos componentes: las elecciones de vida y las oportunidades en la vida; el rango de libertad del individuo para elegir se confronta con su situación en la vida. Las restricciones estructurales pueden ser dominantes. Asignar toda la responsabilidad a la persona puede llegar a ser inapropiado e injusto.

El presente estudio puede tener algunas limitaciones, referidas sustancialmente a la recolección de datos de mortalidad. La cobertura y calidad de los registros de mortalidad en México ha sido considerada como satisfactoria por la Organización Panamericana de la Salud^34^ y ha mejorado en años recientes^35^, lo cual sin dudas avala la consistencia de los datos utilizados. Aun así, vale señalar que pudieran existir dificultades en la codificación de la DM como causa básica de defunción, relacionadas en particular con el hecho de que las personas que padecen la enfermedad fallecen por complicaciones asociadas con enfermedades cardiovasculares o renales, por ejemplo, lo que pudiera en cierta medida distorsionar la verdadera dimensión del problema^23,36^. Esta situación merecería, en sí misma, una investigación más detallada, la cual rebasa los objetivos del presente estudio.

En lo concerniente al método empleado para el cálculo de los AEVP, el uso del supuesto de mortalidad nula entre tres posibles opciones no altera el sentido de los resultados y es recomendado por la literatura^21^ en tanto facilita la interpretación de los resultados y explica en su totalidad los cambios en la EVT.

No obstante las limitaciones enunciadas previamente, los resultados hallados permiten una aproximación razonable al comportamiento reciente de la mortalidad por DM en adultos mayores en México. Dadas las implicaciones que tienen los hallazgos de esta investigación (la DM está reduciendo sustancialmente la EV de la población mexicana, en especial de los adultos mayores y más marcadamente en los hombres), sería recomendable la implementación (o intensificación) de medidas preventivas contra la DM desde edades tempranas, ante la mera sospecha de su potencial aparición, y no solo centradas en quienes han sido diagnosticados. En tal sentido, es necesaria la generación de políticas públicas eficaces que incidan en fomentar estilos de vida saludables, como pueden ser aquellas encaminadas a disminuir el consumo de bebidas azucaradas y las que favorezcan una alimentación adecuada ya desde las escuelas.

Igualmente, es fundamental permitir al paciente que manifieste sus puntos de vista, desde una posición pro-activa y corresponsable, dando oportunidad a identificar los determinantes sociales que limitan la posibilidad de cambios permanentes en su estilo de vida, buscar posibles soluciones y evitar así oportunamente el deterioro de la salud, las comorbilidades y el incremento de los costos de la atención, tanto para la población como para el sistema sanitario.
